# Regulation of ovarian function in domestic animals: translational insights into human ovarian dynamics and dysfunction

**DOI:** 10.3389/fendo.2026.1863960

**Published:** 2026-07-07

**Authors:** Dinesh Dadarwal, Rodrigo A. Carrasco

**Affiliations:** 1 Department of Large Animal Clinical Sciences, Western College of Veterinary Medicine, University of Saskatchewan, Saskatoon, SK, Canada; 2 Department of Veterinary Biomedical Sciences, Western College of Veterinary Medicine, University of Saskatchewan, Saskatoon, SK, Canada

**Keywords:** corpus luteum, domestic animals, farm animals, follicular waves, GnRH, kisspeptin, ovarian aging, ovarian follicle

## Abstract

Ovarian follicular development is a tightly regulated, dynamic process coordinated by endocrine, paracrine, neuroendocrine, and vascular mechanisms that together determine follicle recruitment, selection, dominance, and ovulation. In monovulatory mammals, follicular growth occurs in wave-like patterns characterized by the periodic emergence of a cohort of antral follicles, followed by selection of a single dominant follicle. This process is initiated by transient increases in follicle-stimulating hormone, which drive early follicular recruitment and growth, while subsequent deviation in follicular growth rates leads to dominance of a single follicle capable of continued development. The selection and persistence of the dominant follicle depend on the integration of systemic gonadotropin support with intraovarian growth factor signaling. Local regulatory systems within the ovary, including insulin-like growth factor pathways and oocyte-derived factors such as bone morphogenic protein (BMP15) and growth differentiation factor (GDF9), modulate granulosa cell proliferation, differentiation, and steroidogenic activity. These intraovarian signals establish a functional microenvironment that determines follicular fate and ensures coordinated communication between the oocyte and surrounding somatic cells. A critical but often underappreciated component of follicular maturation is vascular remodeling within the follicular wall. As follicles progress toward preovulatory status, localized increases in blood flow enhance delivery of endocrine signals and metabolic substrates while supporting rapid cellular proliferation and steroid hormone synthesis. These vascular changes are closely associated with follicular health and are predictive of developmental competence and ovulatory potential. At the systemic level, reproductive function is governed by pulsatile secretion of gonadotropin-releasing hormone, which regulates luteinizing hormone release and coordinates ovarian activity. This neuroendocrine axis is modulated by hypothalamic networks that integrate metabolic status, environmental cues, and reproductive signals, thereby linking energy balance with fertility. Emerging evidence from transcriptomic and cellular profiling studies further highlights the complexity of ovarian organization, revealing heterogeneity among follicular and stromal cell populations and identifying novel regulatory pathways involved in follicle activation and aging. Together, these findings emphasize that follicular development is not solely hormone-driven but arises from the integration of endocrine signaling, intraovarian communication networks, vascular dynamics, and neuroendocrine regulation. Disruption of any component of this system contributes to impaired folliculogenesis, reduced oocyte quality, and reproductive dysfunction.

## 1 Introduction

Understanding of cyclic ovarian function in women has been substantially shaped by research in domestic animal species. While rodent models remain indispensable for genetic and mechanistic studies, their reproductive physiology differs fundamentally from that of humans: rodents are polyovulatory, exhibit short estrous cycles, and operate under neuroendocrine rhythms adapted to a distinct reproductive strategy. In contrast, cattle, sheep, and horses are predominantly monovulatory species that closely mirror key features of the human menstrual cycle, including follicular wave dynamics and endocrine organization (1, 2). Pigs, although polyovulatory, have contributed key insights into intraovarian signaling and steroidogenesis (3).

Across domestic species, ovarian function is governed by tightly coordinated endocrine rhythms and intraovarian signaling networks spanning follicular recruitment, selection, ovulation, luteal function, and hypothalamic–pituitary control of gonadotropin secretion (4). The ability to perform longitudinal endocrine profiling combined with real-time ultrasonography has enabled direct temporal mapping of ovarian dynamics *in vivo*, a level of resolution not achievable in most human studies (2, 5). These attributes have established domestic species as foundational translational models in reproductive endocrinology.

A major conceptual advance emerged from bovine ovarian ultrasonography, which demonstrated that folliculogenesis occurs in discrete waves rather than as a continuous process (1, 6). This discovery fundamentally altered the understanding of ovarian follicle recruitment and selection. Subsequent confirmation of follicular wave dynamics in women demonstrated that this organizational principle is conserved in humans, reshaping ovarian stimulation strategies in clinical reproductive medicine (7). A second major advancement was the demonstration that GnRH secretion must be pulsatile to sustain gonadotropin release, establishing the neuroendocrine basis of reproductive cyclicity (8). This principle underpins modern therapeutic GnRH pulse paradigms in hypothalamic infertility and remains a cornerstone of reproductive neuroendocrinology.

More recently, identification of the Kisspeptin, neurokinin B, and dynorphin (KNDy) neuronal network provided a mechanistic framework for GnRH pulse generation. The KNDy-expressing neurons in the arcuate nucleus form an intrinsic oscillatory system integrating steroid feedback and metabolic cues to generate GnRH pulsatility (9, 10). This discovery represents a major unifying advance in hypothalamic control of reproduction. In parallel, genetic studies in sheep identified mutations in bovine morphogenic protein (BMP)15 as determinants of ovulation rate, establishing a causal link between oocyte-derived signaling and follicular outcome (11). This work, together with subsequent identification of growth differentiation factor (GDF)9 function, established oocyte-derived transforming growth factor (TGF)-β signaling as a central regulator of mammalian folliculogenesis and fertility (12).

Despite these advances, mechanistic findings are still often interpreted within species-specific frameworks rather than integrated into a unified model of mammalian ovarian function. Translation therefore relies on analogy rather than formal mechanistic equivalence. A systems-level comparative framework linking follicular, neuroendocrine, intraovarian, and luteal processes across species remains lacking but is essential for improving translational predictability in reproductive medicine.

This review is mechanistic in scope. Domestic animal species are examined as experimental systems for conserved ovarian biology, with emphasis on regulatory principles that are shared across mammals, rather than on species-specific disease phenotypes. Human anovulatory disorders are referenced only when they directly illuminate conserved mechanisms; their treatment as farm-animal disease models falls outside the boundaries of the present review.

We synthesize ovarian regulation across domestic species within four domains: follicular wave dynamics, neuroendocrine regulation of the hypothalamic–pituitary–ovarian axis, intraovarian signaling, and luteal function. Naturally occurring reproductive dysfunctions such as cystic ovarian disease and luteal insufficiency, are as examined specifically for the neuroendocrine and mechanistic insights they provide rather than as integrated disease models. Across all sections, emphasis is placed on conserved mammalian mechanisms with explicit recognition of species-specific adaptations.

## 2 Follicular wave dynamics

### 2.1 Discovery and characterization of follicular waves in cattle

The application of real-time transrectal ultrasonography in cattle led to a fundamental revision of folliculogenesis by demonstrating that antral follicle development occurs in temporally discrete, coordinated cohorts rather than as a continuous recruitment process (1, 6). This established the follicular wave model, in which a synchronized cohort of follicles is recruited and subsequently subjected to a selection process that yields a single dominant follicle (2). Follicular wave emergence is driven by a pituitary rebound in follicular stimulating hormone (FSH) secretion triggered by the decline in estradiol and inhibin that accompanies the onset of dominant follicle regression (13). This endocrine change reflects reduced suppression of pituitary gonadotroph function, leading to increased FSH synthesis and secretion (14). The recruited follicles within a wave depend on FSH for survival and early growth, with granulosa cell proliferation and differentiation mediated through FSH receptor-driven cyclic adenosine monophosphate (cAMP) signaling and induction of aromatase activity (13, 15). As follicular growth proceeds, rising estradiol and inhibin B secretion progressively suppress FSH, producing a declining endocrine environment that constrains further recruitment (2, 12). This feedback is reinforced by increasing inhibin B output from the growing follicular cohort, sharpening competitive divergence among follicles (12).

Follicular fate is determined by integration of gonadotropin signaling with intraovarian regulatory networks rather than endocrine input alone (16). The dominant follicle is distinguished by early acquisition of luteinizing hormone (LH) responsiveness in granulosa cells, allowing continued steroidogenesis under reduced FSH availability (16). This transition is accompanied by remodeling of intracellular signaling pathways, including enhanced cAMP responsiveness and increased steroidogenic enzyme expression (15). Local metabolic regulation via the insulin like growth factor (IGF) system further amplifies gonadotropin sensitivity in the dominant follicle (17). Increased IGF bioavailability, driven by reduced IGF-binding proteins, enhances FSH receptor signaling and supports estradiol production and follicular survival (15, 17). In parallel, oocyte-derived factors such as BMP15 and GDF9 modulate granulosa cell function, including proliferation rate, differentiation state, and gonadotropin sensitivity (12). These signals act in a dose- and stage-dependent manner, contributing to follicular fate decisions within each wave (12, 18). Subordinate follicles fail to maintain this integrated signaling environment and undergo atresia through activation of intrinsic apoptotic pathways and loss of trophic support (15, 19). Dominance therefore arises from coordinated endocrine withdrawal and local amplification of survival signaling, rather than from passive escape from FSH limitation.

### 2.2 Evolutionary diversification of a conserved wave system

The follicular wave architecture is broadly conserved among monovulatory mammals, but species-specific differences arise from quantitative modulation of endocrine thresholds and intraovarian responsiveness (2). In cattle, two to three follicular waves are typically observed per estrous cycle, with ovulation occurring only from the final wave after luteolysis (2). In sheep, reduced strictness in follicular selection allows multiple follicles within a wave to persist longer, increasing ovulation rate through altered sensitivity to oocyte-derived and gonadotropic signals (12, 20). In mares, follicular waves are present but characterized by extended dominance of the preovulatory follicle, reflecting prolonged LH support and sustained IGF activity (2, 21). In pigs, follicular recruitment proceeds with reduced hierarchical suppression, allowing multiple follicles to reach preovulatory size within a compressed endocrine window (3). Across species, variability in ovulation rate reflects shifts in endocrine sensitivity thresholds and intraovarian signaling balance rather than changes in the fundamental wave-generating system.

### 2.3 Follicular waves in women: conserved architecture with variability

In women, longitudinal ovarian ultrasonography demonstrates that follicular development also occurs in discrete wave-like patterns, typically comprising two to three waves per menstrual cycle (7, 22). Each wave is initiated by a transient increase in FSH, which recruits a cohort of antral follicles into coordinated growth (7). However, the expression of waves varies across individuals and cycles, reflecting differences in ovarian reserve status, endocrine milieu, and hypothalamic–pituitary regulation (23). The magnitude of FSH rebound following luteolysis is influenced by luteal progesterone dynamics, which modulate GnRH pulse frequency and downstream gonadotropin secretion (14). Dominant follicle selection occurs when one follicle achieves a relative advantage in FSH receptor expression and estradiol output, enabling suppression of competing follicles (23). This dominance is reinforced by IGF-mediated potentiation of gonadotropin signaling, which enhances granulosa cell proliferation and steroidogenesis (17). Anti-mullerian hormone (AMH) regulates early follicular recruitment by suppressing activation of primordial and early antral follicles through inhibition of phosphoinositide 3-kinase/protein kinase-B (PI3K/AKT) signaling pathways (24, 25). Thus, follicular waves in women emerge from dynamic interaction between systemic gonadotropin oscillations and intraovarian regulatory networks that collectively determine cohort size and competitive outcome.

### 2.4 Implications for assisted reproduction and ovarian reserve

The identification of follicular wave dynamics in domestic species provided the conceptual basis for modern controlled ovarian stimulation strategies (5, 26). In cattle, for instance, synchronization of follicular waves enables precise control of ovulation timing through manipulation of progesterone and gonadotropin environments (26). In human assisted reproduction, controlled ovarian stimulation relies on suppression of endogenous cyclicity combined with exogenous FSH administration to recruit a synchronized follicular cohort (27). Various GnRH analogues are used to prevent premature LH surges that would otherwise trigger luteinization and disrupt coordinated follicular development (27). The circulating levels of AMH reflect functional activity of small growing follicles and contributes to regulation of early follicular recruitment, linking endocrine measurement to underlying ovarian physiology (25). Antral follicle count provides a structural estimate of recruitable follicles at a given time but does not capture dynamic wave behavior or selection kinetics (28). Despite clinical utility, a mechanistic discontinuity persists between static ovarian reserve markers and the dynamic processes governing follicular wave emergence, dominance, and oocyte competence (28).

### 2.5 Follicular vascularization and oocyte competence

Follicular growth beyond the early antral stage requires the promotion of perifollicular angiogenesis to sustain the metabolic and endocrine demands of proliferating granulosa and theca cells. Vascular endothelial growth factor (VEGF) is a protein member of the platelet-derived growth factor family that is upregulated in ovarian tissues through gonadotropin-stimulated cAMP/PKA signaling and hypoxia-mediated hypoxia-inducible factor (HIF)-1α activation, driving endothelial proliferation, migration, and tubulogenesis within the theca layer (29–32). Angiopoietin-1 and angiopoietin-2 acting through tyrosine kinase (Tie2) receptors subsequently coordinate vessel stabilization and remodeling, balancing endothelial quiescence with the structural plasticity required for continued angiogenic expansion (29, 33). The VEGF isoform-specific contributions to follicular angiogenesis and the role of lymphatic vessels in follicular fluid homeostasis remain incompletely characterized in both cattle and women.

In cattle, perifollicular blood flow diverges between dominant and subordinate follicles concurrently with follicular selection, positioning enhanced vascularization as a mechanistic contributor to dominance rather than its consequence (34–36). Following the preovulatory LH surge, acute vascular remodeling within the follicular wall is driven by locally released prostaglandins and vasoactive peptides acting as vasodilatory mediators during final oocyte maturation (37–39). Perifollicular vascular perfusion at ovum pick-up correlates positively with follicular steroidogenic output, fertilization rates, and blastocyst development in heifers, directly linking the follicular vascular microenvironment to oocyte developmental competence (35, 40).

Intrafollicular oxygen tension is a critical mechanistic intermediary between perifollicular perfusion and oocyte quality. In cattle, oxygen availability during follicular maturation directly governs oocyte gene expression, mitochondrial activity, and embryo development (41, 42). Insufficient intrafollicular oxygen compromises mitochondrial ATP synthetic capacity and disrupts perinuclear mitochondrial distribution in bovine oocytes, impairing cytoplasmic maturation, meiotic spindle assembly, and chromosome segregation (43). In women, follicular fluid oxygen content correlates positively with perifollicular vascularity and intrafollicular VEGF concentrations, with higher dissolved oxygen predicting superior fertilization and developmental outcomes, establishing oxygen tension as a conserved determinant of oocyte competence (44, 45).

In human IVF, power Doppler quantification of perifollicular blood flow correlates independently with follicular steroidogenic output, oocyte recovery, fertilization competence, and embryo quality, confirming that the follicular vascular microenvironment carries forward into oocyte developmental potential (46–48). However, variability in predictive strength across studies likely reflects differences in assessment timing relative to the human chorionic gonadotropin (hCG) trigger and methodological inconsistencies in Doppler quantification. In poor ovarian responders to superstimulation, perifollicular vascular insufficiency reduces gonadotropin bioavailability at the granulosa cell level, compromises steroidogenic substrate delivery, and diminishes intrafollicular oxygen tension, collectively impairing oocyte maturation (49, 50). In contrast, pharmacological enhancement of follicular perfusion through nitric oxide pathway activation and phosphodiesterase-5 inhibition partially restores perifollicular blood flow and improves oocyte quality in this population (50, 51). Whether standardized perifollicular vascular assessment could serve as an independent biomarker of oocyte competence beyond conventional endocrine and morphometric markers, and how follicular vascular insufficiency interacts with ovarian reserve decline at the molecular level, remain unanswered questions with direct implications for optimizing ovarian stimulation protocols.

## 3 Neuroendocrine aspects of ovarian function

### 3.1 The hypothalamic–pituitary–ovarian axis: conserved architecture and pulsatile GnRH secretion

**Figure 1:**
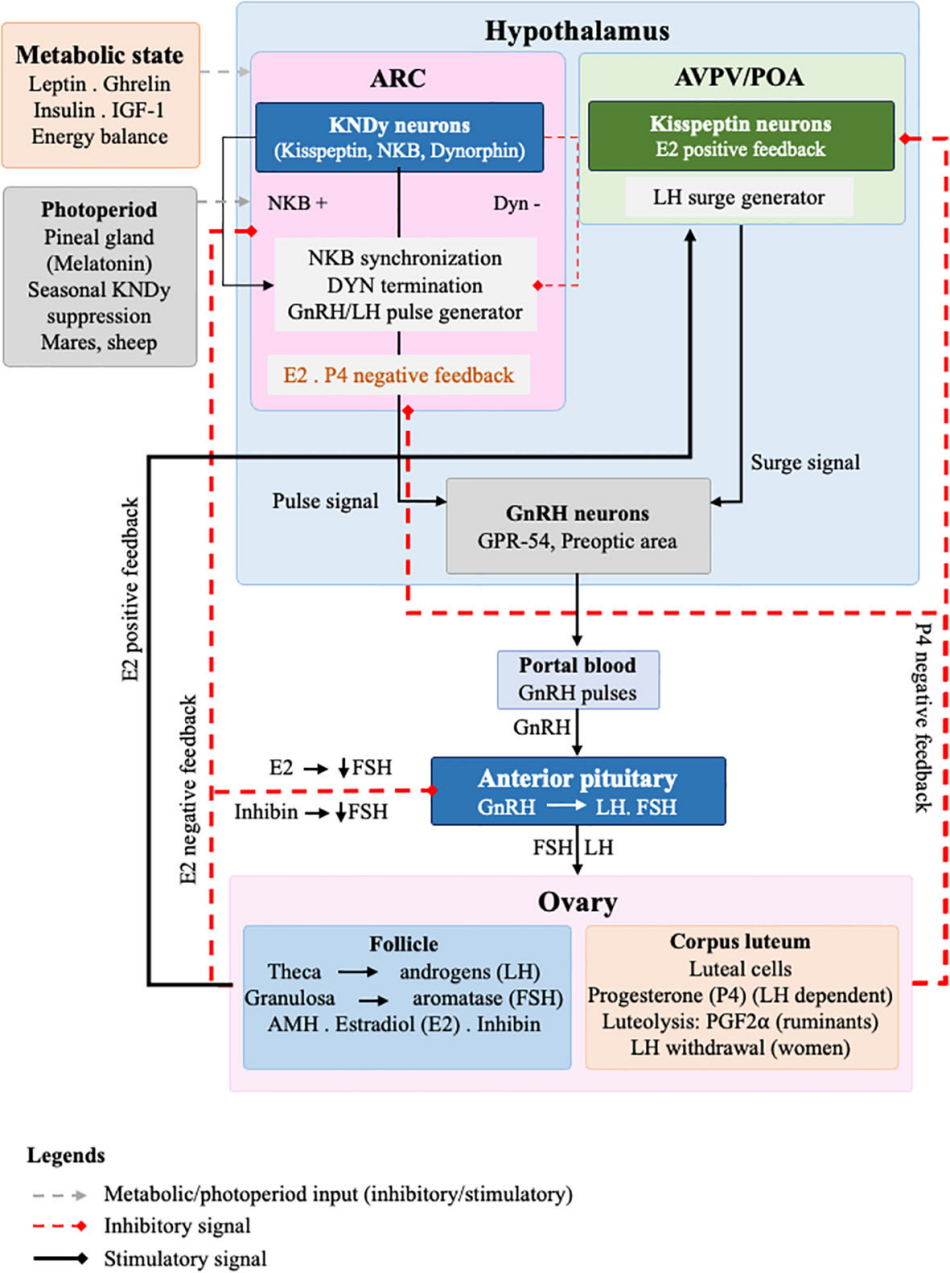
Schematic organization of the hypothalamic-pituitary-ovarian (HPO) axis with KNDy circuitry and steroid feedback regulation across mammalian species. The HPO axis operates as a closed-loop neuroendocrine system governing gonadotropin secretion and ovarian function. KNDy neurons in the arcuate nucleus generate pulsatile GnRH release through NKB-driven network synchronization and dynorphin-mediated termination, a mechanism conserved across cattle, sheep, horses, pigs, and women. Species differences arise in GnRH pulse frequency and amplitude rather than oscillatory architecture. AVPV/POA kisspeptin neurons mediate estradiol positive feedback and the preovulatory LH surge. GnRH is delivered to pituitary gonadotrophs via the portal blood system in a pulsative fashion that is critical for promoting gonadotrophin secretion; continuous GnRH exposure causes receptor desensitization across all species. LH and FSH drive two-cell steroidogenesis in the ovarian follicle, a mechanism conserved across species with quantitative differences in IGF amplification accounting for ovulation rate variation. Luteal progesterone secretion is LH-dependent across species; luteolysis is PGF2α-driven in ruminants but involves LH withdrawal and immune-mediated regression in women, with conserved terminal intracellular endpoints. Metabolic inputs via leptin, ghrelin, insulin, and IGF-1 converge on arcuate KNDy networks, linking energy balance to reproductive function in cattle, sheep, and women. Photoperiodic suppression via melatonin is present in sheep and mares but absent in cattle and women. Black solid arrows-stimulatory signals; red dashed arrows with diamond end – inhibitory/suppression pathways; grey dashed arrow – metabolic or photoperiod input. HPO, hypothalamic-pituitary-ovarian; KNDy, kisspeptin/neurokinin B/dynorphin; GnRH, gonadotropin-releasing hormone; NKB, neurokinin B; AVPV/POA, anteroventral periventricular/preoptic area; LH, luteinizing hormone; FSH, follicle-stimulating hormone; E_2_, estradiol; P_4_, progesterone; PGF2α, prostaglandin F2α; IGF, insulin-like growth factor; AMH, anti-Müllerian hormone.

Reproductive function in female mammals is governed by the hypothalamic–pituitary–ovarian (HPO) axis, a hierarchically organized neuroendocrine system in which hypothalamic neurons regulate pituitary gonadotropin secretion and ovarian outputs feedback to upstream centers. Rather than functioning as a linear pathway, the HPO axis operates as a closed-loop dynamical system in which ovarian steroids, peptides, and metabolic signals continuously modulate hypothalamic and pituitary activity (52, 53). The core components are conserved across mammalian species: GnRH neurons, anterior pituitary gonadotrophs secreting LH and FSH, and ovarian follicles and corpora lutea acting as both endocrine targets and feedback regulators (**Figure 1**). Reproductive diversity across species arises primarily from differences in the temporal patterning of GnRH secretion and sensitivity to steroid feedback rather than structural divergence of the axis itself.

A defining characteristic of this axis is its dependence on pulsatile GnRH secretion. Continuous GnRH exposure induces pituitary GnRH receptor desensitization and downregulation, resulting in suppression of LH and FSH secretion, whereas pulsatile stimulation maintains gonadotroph responsiveness and preserves cyclical ovarian function (8, 54). This requirement reflects intrinsic GnRH receptor signaling kinetics and downstream gonadotroph transcriptional responsiveness (55). The arcuate nucleus serves as the site of GnRH pulse generation, with its neuronal networks receiving and integrating steroid feedback and nutritional status signals. That ovarian steroids exert tonic suppression of the axis is demonstrated by the rise in GnRH and LH pulsatile activity observed following ovariectomy (52, 53). Progesterone reduces GnRH pulse frequency primarily via hypothalamic inhibitory interneurons, including gamma-aminobutyric acid (GABA)ergic and opioid-mediated pathways, whereas estradiol exerts both negative and positive feedback depending on concentration and exposure duration (52, 56). Estrous cyclicity therefore reflects alternating phases of progesterone-dominant suppression during the luteal phase and estradiol-driven positive feedback following luteolysis, culminating in the preovulatory LH surge driven by hypothalamic network reconfiguration rather than pituitary-only activation.

### 3.2 Steroid feedback regulation and KNDy control of GnRH output

Ovarian steroids exert bidirectional control over GnRH and gonadotropin secretion through anatomically and functionally distinct hypothalamic circuits. Estradiol at low-to-moderate concentrations suppresses GnRH and LH secretion via estrogen receptor (ER)α-expressing neurons in the arcuate nucleus and preoptic area (52), while progesterone reinforces this inhibitory tone in ruminants through GABAergic and dynorphin-mediated opioid pathways (53). Sustained high estradiol concentrations switch hypothalamic output to positive feedback mode, culminating in the preovulatory LH surge. This surge depends on coordinated activation of kisspeptin neurons in the anteroventral periventricular/preoptic region (AVPV/POA), where estradiol-responsive kisspeptin neurons act as a relay between steroid feedback and GnRH neuron activation (9, 57). The discovery of kisspeptin signaling and its receptor G-protein coupled receptor (GPR)54 (KISS1R) established a fundamental upstream regulatory system controlling GnRH secretion and fertility (58, 59). Loss-of-function mutations in this pathway result in hypogonadotropic hypogonadism, confirming its essential physiological role. Two anatomically and functionally distinct kisspeptin populations exist in domestic species: AVPV/POA neurons mediating estradiol-positive feedback and arcuate neurons forming the KNDy network responsible for GnRH pulse generation. Arcuate KNDy neurons co-express kisspeptin, neurokinin B (NKB), and dynorphin and function as an intrinsic oscillatory network. The NKB acts as a synchronizing excitatory signal via tachykinin (TACR3) receptors, whereas dynorphin acts via κ-opioid receptors to terminate network activity, generating rhythmic oscillations underlying pulsatile GnRH secretion (9, 10, 57).

Experimental disruption of KNDy signaling abolishes LH pulsatility, while pharmacological activation of kisspeptin or neurokinin pathways restores pulsatile gonadotropin secretion, confirming that KNDy neurons function as the central hypothalamic pulse generator (10, 35). More recent circuit-level studies indicate that KNDy neurons integrate steroidal, metabolic, and environmental inputs, positioning them as a distributed neuroendocrine oscillator rather than a simple relay system. AVPV/POA kisspeptin neurons, in contrast, primarily mediate the estradiol-induced LH surge, demonstrating functional specialization within the kisspeptin system (60).

### 3.3 Metabolic and nutritional modulation of the HPO axis

Reproductive function is highly sensitive to metabolic state, with energy availability acting as a primary integrative signal linking homeostatic status to hypothalamic GnRH output. In ewes and cattle, negative energy balance suppresses LH pulse frequency, delays puberty, and prolongs postpartum anestrus (61, 62). These effects originate at the hypothalamic level and converge on KNDy and kisspeptin networks, which act as metabolic sensors of reproductive viability. Key metabolic signals include leptin, insulin, ghrelin, and glucose availability. Leptin deficiency reduces kisspeptin expression in the arcuate nucleus and diminishes GnRH pulse frequency, while ghrelin and energy deficit-associated neuropeptides exert inhibitory effects on KNDy neuron activity (63, 64). Insulin and IGF-1 act as permissive metabolic signals, modulating hypothalamic sensitivity and ovarian steroidogenesis, thereby linking systemic energy status to both central and peripheral reproductive control (61).

### 3.4 Seasonal regulation and photoperiodic control

Seasonally breeding species such as sheep and horses provide a robust physiological model of reversible hypothalamic reproductive suppression. Photoperiodic information is transduced via melatonin secretion from the pineal gland, which modulates hypothalamic reproductive circuits. During the non-breeding season, increased sensitivity to estradiol suppresses kisspeptin expression in both arcuate and preoptic regions (65, 66), leading to reduced GnRH pulse frequency and functional suppression of the HPO axis. Importantly, the LH surge mechanism remains intact during anestrus. Exogenous estradiol still induces an LH surge of comparable magnitude across breeding and non-breeding seasons, indicating that reproductive suppression reflects reduced GnRH pulsatility rather than loss of positive feedback capacity (66). Return to the breeding season involves sequential reactivation of kisspeptin neurons, reduction of inhibitory opioid tone, and restoration of GnRH pulsatility. This represents reversible network-level neuroplasticity rather than structural neuronal change. Comparative evidence suggests partial mechanistic overlap between seasonal anestrus in domestic animals and functional hypothalamic amenorrhea in women, where suppression of GnRH pulsatility is mediated by reduced kisspeptin drive, increased inhibitory tone, and altered steroid feedback sensitivity converging on KNDy network dysfunction (62, 66, 67).

## 4 Intraovarian signaling and follicle selection

### 4.1 Oocyte–somatic cell communication and conserved determinants of follicular competence

Rather than passively receiving cues from surrounding somatic cells, the mammalian oocyte is an active participant in follicular regulation, exchanging signals with granulosa and cumulus cells through gap junction channels at transzonal projection contacts and through paracrine factor secretion into the follicular fluid (68, 69). Bovine models have been instrumental in defining these processes due to the accessibility of developmentally staged follicles. Experimental studies demonstrate that the oocyte suppresses premature luteinization of granulosa cells, promotes glycolytic metabolism in cumulus cells to support oocyte oxidative phosphorylation, and regulates granulosa proliferation and differentiation. These effects are mediated largely through oocyte-secreted TGF-β superfamily ligands, particularly GDF9 and BMP15, which act in cooperative, stage-dependent fashion (70, 71). In addition, oocyte-derived factors regulate granulosa cell FSH receptor expression and responsiveness, ensuring that follicular sensitivity to gonadotropins is dynamically modulated during growth (15, 72). This establishes the follicle as a functional signaling unit in which developmental competence arises from reciprocal oocyte–somatic communication rather than unilateral endocrine control.

A further regulatory layer is provided by AMH expression in granulosa cells of small antral follicles, which limits premature FSH-dependent recruitment and contributes to hierarchical follicle organization (24, 25). Both GDF9 and BMP15 are oocyte-derived TGF-β superfamily members essential for regulating granulosa cell proliferation, differentiation, and FSH responsiveness. In sheep, heterozygous mutations in BMP15 (FecX) and GDF9 (FecG) increase ovulation rate by reducing granulosa cell sensitivity to paracrine inhibition, whereas homozygous mutations result in follicular arrest and infertility, establishing gene-dosage-dependent control of ovulation rate and follicular hierarchy (11, 73). In women, rare variants in BMP15 and GDF9 are associated with primary ovarian insufficiency and altered ovarian reserve, supporting functional conservation across species (74, 75).

At the molecular level, BMP15 and GDF9 form highly bioactive heterodimers (cumulin) that exhibit greater potency than homodimers in stimulating granulosa cell function and cumulus expansion (76). Cumulus expansion is driven through downstream induction of hyaluronan synthesis pathways and EGF-like growth factor signaling, integrating oocyte-derived cues with gonadotropin action to coordinate oocyte maturation (77). These findings position oocyte-derived TGF-β signaling as a central determinant of follicular fate and ovulatory efficiency.

### 4.2 Granulosa–theca cell steroidogenic cooperation and follicular endocrine specialization

**Figure 2:**
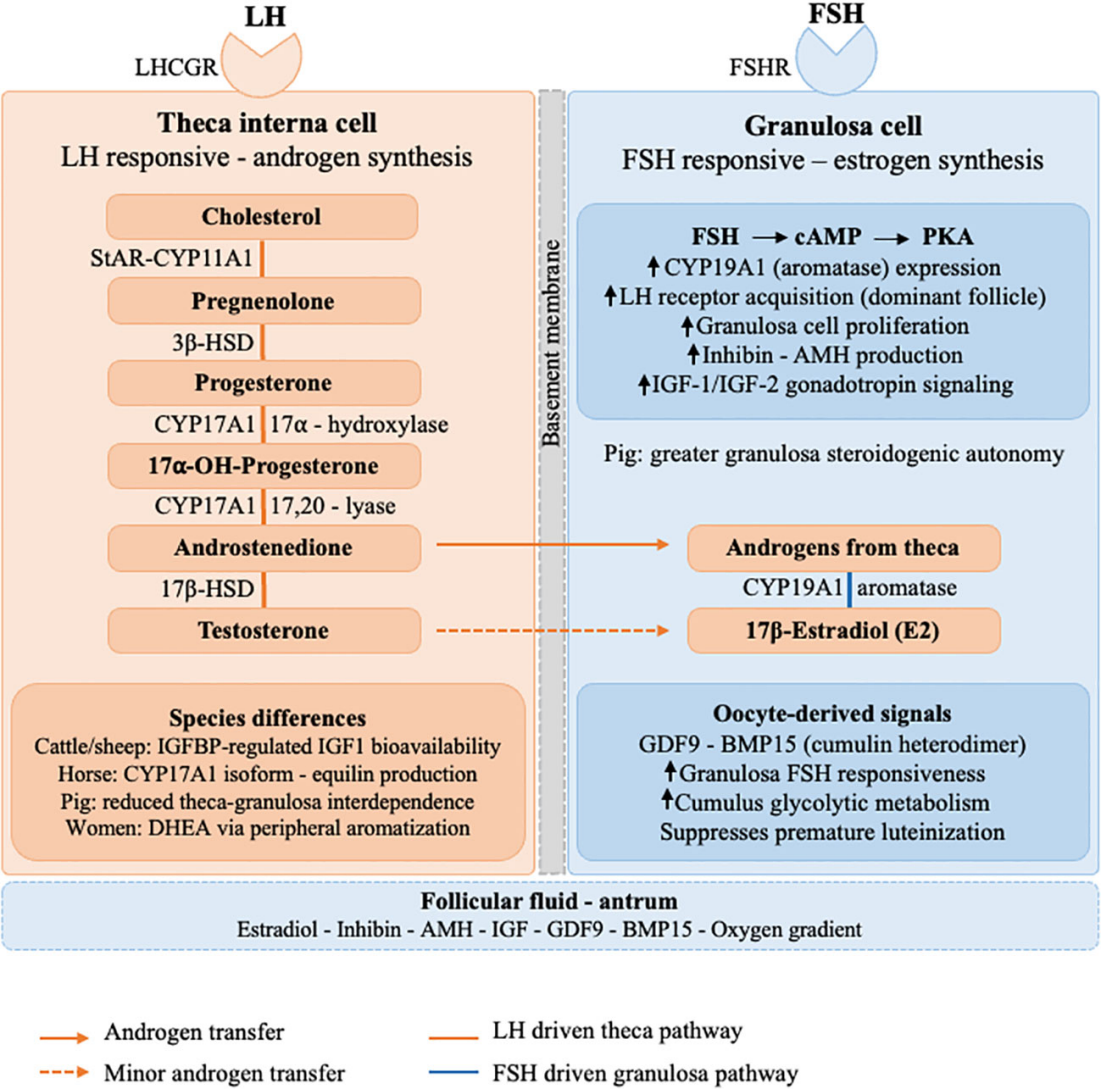
Two-cell, two-gonadotropin model of ovarian follicular steroidogenesis across mammalian species. Theca interna cells respond to LH through LHCGR-coupled cAMP/PKA signaling, driving a conserved androgen biosynthetic cascade. StAR-mediated cholesterol transport into mitochondria enables CYP11A1-catalyzed side-chain cleavage to pregnenolone; 3β-HSD converts pregnenolone to progesterone; and CYP17A1 sequentially performs 17α-hydroxylation and 17,20-lyase activities to generate androstenedione and testosterone. Both androgens diffuse across the avascular basement membrane into the granulosa compartment, where FSH acting through FSHR-coupled cAMP/PKA signaling induces CYP19A1 expression and aromatization to 17β-estradiol. Beyond steroidogenesis, FSH drives LH receptor acquisition in granulosa cells of the dominant follicle, the molecular event marking the transition from FSH-dependent to LH-responsive growth, and stimulates granulosa cell proliferation and inhibin B and AMH secretion. IGF-1 and IGF-2 amplify gonadotropin signaling in both cell compartments, with IGFBP-regulated bioavailability accounting for the primary species variation in IGF activity. Oocyte-derived GDF9 and BMP15, acting cooperatively as the cumulin heterodimer through TGF-β/SMAD signaling, regulate granulosa FSH responsiveness, redirect cumulus cell metabolism toward glycolysis to support oocyte oxidative phosphorylation, and suppress premature luteinization. The two-cell architecture is conserved across cattle, sheep, horses, pigs, and women; species divergence is quantitative, arising in IGFBP regulation, CYP17A1 isoform activity, and the degree of granulosa steroidogenic autonomy rather than in pathway organization. Solid arrows, enzymatic conversion or stimulatory signals; dashed arrows, minor transfer pathway; dashed border, basement membrane. StAR, steroidogenic acute regulatory protein; CYP11A1, cholesterol side-chain cleavage enzyme; 3β-HSD, 3β-hydroxysteroid dehydrogenase; CYP17A1, 17α-hydroxylase/17,20-lyase; CYP19A1, aromatase; LH, luteinizing hormone; FSH, follicle-stimulating hormone; LHCGR, LH/choriogonadotropin receptor; FSHR, FSH receptor; IGF, insulin-like growth factor; IGFBP, IGF-binding protein; GDF9, growth differentiation factor 9; BMP15, bone morphogenetic protein 15; AMH, anti-Müllerian hormone; E2, 17β-estradiol; DHEA, dehydroepiandrosterone; TGF-β, transforming growth factor-β.

Ovarian estradiol synthesis is governed by the two-cell, two-gonadotropin model in which coordinated theca–granulosa interactions produce steroid hormones essential for follicular development and ovulation (**Figure 2**). Theca interna cells respond to LH and express cytochrome P450 family (CYP11A1 and CYP17A1), enabling androgen synthesis, while granulosa cells aromatize these androgens via FSH-induced CYP19A1 expression. This compartmentalized steroidogenic system integrates endocrine and paracrine signals to ensure follicular maturation aligns with systemic gonadotropin dynamics (78, 79). A key mechanistic feature of follicle selection is FSH receptor upregulation in the dominant follicle, which increases sensitivity to declining FSH concentrations and enables continued growth under endocrine constraint (13). This is reinforced by enhanced LH receptor acquisition in granulosa cells of the dominant follicle, marking the transition from FSH-dependent to LH-responsive steroidogenesis (80).

The IGF system acts synergistically with gonadotropins: IGF-1 enhances FSH signaling via IGF-1 receptor activation and reduced IGF-binding protein sequestration, amplifying granulosa cell proliferation and steroidogenesis (81, 82). Disruption of this system, through excess LH stimulation, intrinsic theca cell hyperactivity, or reduced granulosa aromatase activity, leads to androgen-dominant follicular environments that impair oocyte maturation and follicle selection. These mechanisms are central to hyperandrogenic anovulatory disorders, where disruption of the balance between androgen and estradiol production impairs follicle selection and ovulatory function. Following the preovulatory LH surge, rapid luteinization is initiated through induction of StAR and upregulation of CYP11A1, marking the transition from estradiol to progesterone synthesis (83, 84). This transition is accompanied by cessation of aromatase expression and reprogramming of granulosa cell fate toward luteal differentiation.

### 4.3 Follicle selection, dominance, and oocyte competence

Follicle selection emerges from a self-reinforcing interplay between endocrine withdrawal and intraovarian amplification. The dominant follicle increases estradiol and inhibin secretion, suppressing circulating FSH below the threshold required by subordinate follicles. This endocrine constraint is coupled with acquisition of relative gonadotropin independence through increased granulosa cell LH receptor expression, enabling continued growth despite declining FSH support (13, 70). A complementary intraovarian mechanism is the suppression of atresia through survival signaling pathways (PI3K/AKT, BCL-2 family balance), which distinguishes dominant from subordinate follicles at the cellular level (82). Concurrently, IGF-1 enhances FSH signaling sensitivity in the dominant follicle through reduced IGF-binding protein availability, potentiating granulosa cell proliferation and steroidogenesis while subordinate follicles become increasingly growth-restricted (81, 82). Importantly, AMH expression in smaller follicles contributes to this selection process by limiting premature FSH responsiveness, reinforcing hierarchical recruitment (24). This creates a feed-forward loop in which endocrine suppression and intraovarian sensitization act synergistically to ensure single-follicle dominance in monovulatory species.

Oocyte competence is established in parallel with follicular dominance. In cattle, granulosa and cumulus cell transcriptomic profiles obtained during ovum pick-up correlate strongly with subsequent blastocyst development, demonstrating that somatic cell function reflects oocyte developmental potential (85, 86). Following fertilization, embryonic signaling ensures continuation of luteal function during the pre-implantation period through species-specific molecular mechanisms, interferon-tau in cattle, conceptus-derived estrogens in pigs, and chorionic gonadotropin in humans, all converging on sustained progesterone production (87, 88).

## 5 Luteal regulation

### 5.1 Corpus luteum formation, maintenance, and luteolysis

The corpus luteum (CL) is a transient endocrine gland formed through luteinization of the post-ovulatory follicle, involving granulosa and theca cell trans-differentiation into large and small luteal cells, extensive angiogenesis, and acquisition of high steroidogenic capacity. Progesterone secretion increases rapidly under the trophic influence of LH, which remains essential for luteal maintenance in most domestic species and humans (83, 84). Luteal steroidogenesis depends on acute and chronic LH signaling through cAMP/PKA-mediated induction of steroidogenic acute regulatory protein (StAR) and cholesterol transport into mitochondria, establishing progesterone biosynthesis as a tightly LH-dependent output. Sustained luteal function is reinforced by local angiogenic support (VEGF-mediated vascularization) and autocrine/paracrine luteotropic factors (83).

Luteal lifespan is governed by the balance between luteotropic support (primarily LH) and luteolytic signaling driven by uterine prostaglandin F2α (PGF2α). In ruminants, pulsatile uterine PGF2α release triggers corpus luteum regression through FP receptor-mediated signaling cascades that progressively dismantle steroidogenic capacity: LH receptor numbers decline, cAMP production is impaired, steroidogenic enzymes are downregulated, mitochondria lose function, and apoptotic cell death programs are activated (89, 90). Luteal sensitivity to PGF2α is developmentally regulated. Early and mid-cycle CLs exhibit resistance due to robust steroidogenic output, high intracellular cAMP buffering capacity, and local luteotropic factors including PGE2. In contrast, late-cycle CLs acquire heightened prostaglandin responsiveness as LH support declines, enabling synchronized regression (89, 91). In women, luteolysis is less strictly prostaglandin-driven and involves functional withdrawal of LH support combined with local immune-cell infiltration, cytokine signaling, and tissue remodeling processes. Nevertheless, conserved intracellular endpoints, reduced StAR expression, loss of mitochondrial steroidogenic capacity, oxidative stress, and apoptosis, are shared across species, indicating a conserved terminal luteolytic program despite divergent initiating signals (83, 84).

### 5.2 Luteal progesterone secretion and translational models of luteal phase dysfunction

Progesterone secretion from the CL is essential for endometrial receptivity, embryo implantation, and early pregnancy maintenance. In cattle, insufficient early luteal progesterone impairs endometrial gene expression, conceptus elongation, and embryonic survival, establishing a direct mechanistic link between luteal output and uterine developmental programming (92). Progesterone regulates uterine receptivity through modulation of endometrial epithelial proliferation, glandular secretion, immune tolerance pathways, and histotroph composition. Even modest reductions in early luteal progesterone alter interferon-stimulated gene expression and impair conceptus–maternal signaling in ruminants, underscoring the narrow endocrine threshold required for successful pregnancy establishment (87, 93).

Exogenous progesterone supplementation during early gestation in livestock modifies endometrial transcriptomic profiles, nutrient transport systems, and uterine gland activity, demonstrating that uterine receptivity is highly sensitive to both timing and amplitude of progesterone exposure (94). These findings underpin clinical luteal support strategies in assisted reproductive technologies. In high-producing dairy cows, increased hepatic steroid metabolism reduces circulating progesterone during early lactation, providing a physiological model of metabolically induced luteal insufficiency relevant to energy-deficient reproductive states in women (91).

Experimental reduction of LH pulse frequency in cattle directly impairs luteal steroidogenic capacity through cAMP/PKA-mediated downregulation of StAR expression and cholesterol mobilization, producing smaller CLs with diminished progesterone output and abbreviated lifespan (91, 94). Progressive loss of luteal LH receptor sensitivity further amplifies this functional decline, establishing a feed-forward mechanism through which gonadotropin withdrawal accelerates luteal regression (95). In ewes, premature gonadotropin-induced ovulation produces luteal insufficiency through inadequate preovulatory LH priming, demonstrating that the steroidogenic competence of the CL is determined by the endocrine environment during final follicular maturation rather than solely by postovulatory gonadotropin support (96). In goats, subpopulation-specific differences in luteal cell cholesterol availability and utilization represent a rate-limiting determinant of progesterone synthetic capacity across luteal phase stages, identifying intracellular steroidogenic substrate delivery as a critical regulatory node (97). Across ruminant species, subnormal luteal function therefore reflects mechanistically convergent impairments in gonadotropin receptor signaling, steroidogenic enzyme activity, and substrate availability (98). In women, the clinical construct of luteal phase deficiency has been subject to sustained scrutiny, with poor concordance between endocrine and histological diagnostic criteria and inconsistent reproductive outcomes undermining its validity as a discrete pathological entity (99–102). This diagnostic ambiguity does not diminish the translational utility of ruminant luteal models, which position luteal insufficiency as a physiological continuum of impaired HPO axis signaling rather than a binary pathological state, directly informing ART luteal support strategies including GnRH-induced accessory corpus luteum formation, hCG supplementation, and exogenous progesterone administration (91, 92, 103).

## 6 Ovarian dysfunction: animal models and human parallels

### 6.1 Cystic ovarian disease in cattle

Cystic ovarian disease (COD) in cattle is characterized by the persistence of large anovulatory follicular structures that fail to undergo luteinization or ovulation and persist beyond the normal follicular growth phase in the absence of a functional corpus luteum. Clinically, affected animals exhibit prolonged anestrus, disrupted estrous behavior, and reduced fertility. At the mechanistic level, COD reflects disruption of the integrated HPO axis. A central defect is impaired generation of the preovulatory LH surge, arising from altered hypothalamic sensitivity to estradiol positive feedback, reduced kisspeptin activation, and dysregulated KNDy neuronal output (52, 53). This impairment is closely linked to disrupted GnRH pulse generation and abnormal integration of steroid feedback within arcuate KNDy networks. These neuroendocrine abnormalities are frequently compounded by metabolic stressors such as negative energy balance and insulin resistance, which further suppress GnRH pulsatility and destabilize follicular maturation through altered leptin, insulin, and IGF signaling pathways (61, 62). Concurrently, intraovarian alterations, including increased androgen-to-estradiol ratios, dysregulated theca cell steroidogenesis, and impaired granulosa cell differentiation, contribute to follicular persistence and failure of ovulation. This integrated endocrine–metabolic pathology makes COD a highly informative large-animal model for understanding anovulatory disorders in which hypothalamic dysfunction, metabolic imbalance, and ovarian steroidogenic disruption converge.

### 6.2 Androgen excess and developmental programming of reproductive dysfunction

Prenatal androgen exposure models in sheep provide a powerful experimental framework for understanding how early-life endocrine environments program long-term reproductive dysfunction. Exposure to excess androgens during critical windows of fetal development results in adult phenotypes characterized by increased LH pulse frequency, impaired estradiol and progesterone negative feedback sensitivity, insulin resistance, and defective follicle selection. At the neuroendocrine level, the primary focus of this section, prenatally androgenized ewes exhibit altered hypothalamic organization of kisspeptin and KNDy neuronal networks, leading to impaired LH surge generation and disrupted cyclicity. The density and distribution of kisspeptin neurons in the arcuate nucleus and anteroventral periventricular region are altered by prenatal testosterone exposure, reducing the capacity for appropriate estradiol-positive feedback responses (104–106). These neuroendocrine changes persist into adulthood, demonstrating that androgen excess can permanently reprogram hypothalamic feedback circuits through developmental plasticity of GnRH pulse-generating networks. This provides a mechanistic basis for hyperandrogenic anovulatory disorders in women.

The persistence of neuroendocrine dysfunction following prenatal androgen excess reflects stable epigenetic reprogramming of hypothalamic and ovarian regulatory circuits rather than continued androgen exposure. In prenatally androgenized sheep, excess testosterone induces locus-specific DNA methylation changes at promoter regions governing steroidogenic enzyme and gonadotropin receptor gene expression in the ovary, with concurrent alterations in histone lysine demethylase occupancy that shift chromatin from transcriptionally permissive to repressive configurations at key reproductive regulatory loci (107–109). These chromatin-level modifications establish a molecular memory of early androgen exposure that persists through subsequent cell divisions, maintaining altered transcriptional states in ovarian somatic cells independent of circulating androgen concentrations in adulthood. At the hypothalamic level, epigenetic modification of kisspeptin regulatory regions through promoter DNA methylation and repressive histone marks attenuates kisspeptin transcriptional output from arcuate KNDy neurons, reducing the amplitude and frequency of GnRH pulse generator activity and impairing the capacity for estradiol-positive feedback-driven LH surge generation (110, 111). The convergence of ovarian and hypothalamic epigenetic reprogramming through a common androgen-driven mechanism positions prenatal androgen excess as a developmental event that encodes reproductive dysfunction through durable chromatin architectural changes across multiple levels of the HPO axis.

### 6.3 Luteal phase dysfunction: translational insights from livestock models

The translational importance of large-animal luteal models extend beyond progesterone output to the precise temporal coordination between luteal secretory activity and stage-specific embryonic developmental requirements. In cattle, the endometrial transcriptomic response to progesterone is temporally gated, with early luteal progesterone exposure programming uterine glandular secretory activity and histotroph composition within a narrow developmental window that directly determines conceptus elongation capacity and embryo survival before implantation is established (92, 93). Progesterone-dependent regulation of interferon-stimulated gene expression in the endometrium establishes the molecular framework for conceptus-maternal recognition signaling, with even modest temporal displacement of progesterone rise related to conceptus development impairing this communication axis and embryonic survival (87). These mechanistic relationships, replicated in sheep through controlled embryo transfer studies manipulating recipient luteal age and progesterone environment, demonstrate that uterine receptivity reflects the integration of progesterone concentration, exposure timing, and conceptus developmental stage rather than progesterone amplitude alone. Collectively, these observations illustrate a direct translational continuum from mechanistic livestock studies to clinical assisted reproductive technology protocols aimed at optimizing luteal phase support and improving implantation success (91).

## 7 Reproductive aging and ovarian reserve

### 7.1 Primordial follicle pool dynamics and endocrine hallmarks of ovarian aging

**Figure 3:**
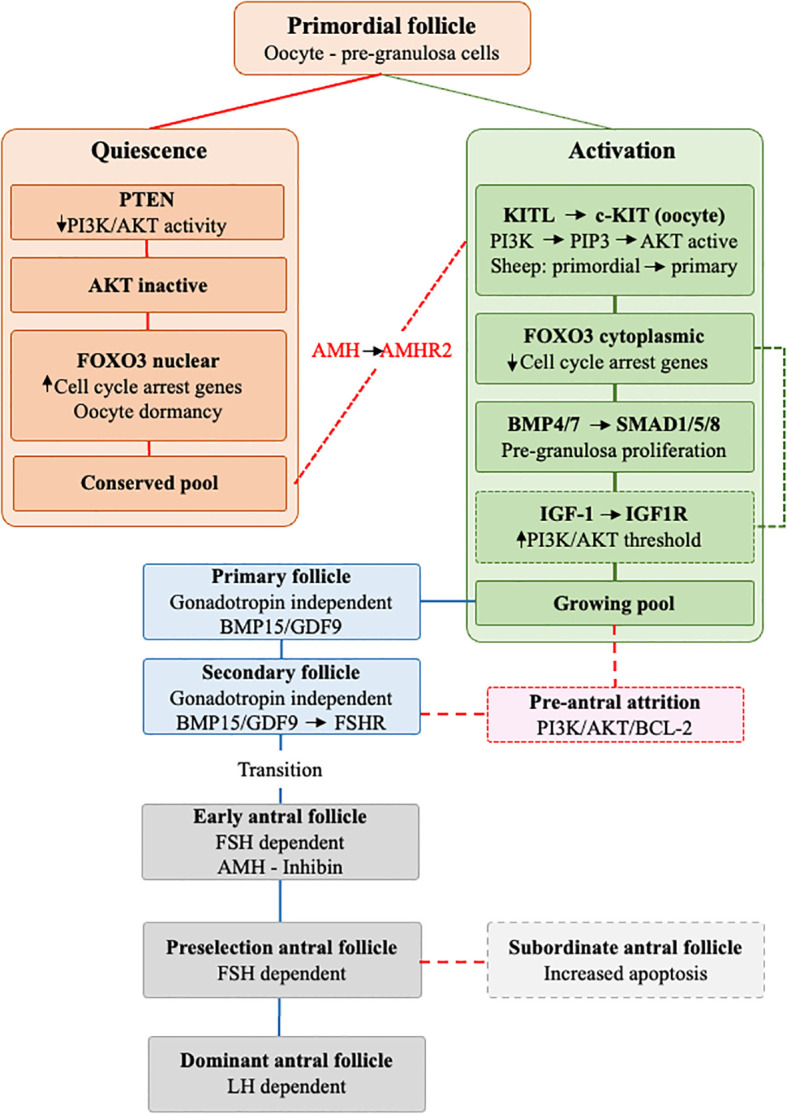
Primordial follicle activation and the gonadotropin-independent to gonadotropin-dependent transition. The primordial follicle pool is maintained in quiescence through PTEN-mediated suppression of PI3K/AKT activity, retaining FOXO3 in its nuclear state where it drives cell cycle arrest gene expression and sustains oocyte dormancy. Two anatomically distinct activation signals converge to initiate follicular recruitment: KIT ligand acting through c-KIT on the oocyte surface drives PI3K-mediated PIP3 generation, activating AKT and mediating FOXO3 cytoplasmic sequestration to relieve cell cycle arrest; and BMP4/BMP7 activates SMAD1/5/8 in pre-granulosa cells to drive somatic cell proliferation and enclosure. IGF-1 acting through IGF1R amplifies PI3K/AKT signaling to establish a self-reinforcing activation threshold. Once initiated, follicular entry into the growing pool is irreversible. AMH, produced by granulosa cells of small growing follicles, acts through AMHR2 to calibrate the activation rate to the remaining ovarian reserve, connecting the growing follicle cohort to the quiescent pool through negative feedback. Activated follicles progress through gonadotropin-independent primary and secondary preantral stages; oocyte-derived GDF9 and BMP15 drive FSH receptor acquisition in granulosa cells, establishing the molecular prerequisite for the transition to gonadotropin-dependent growth at the early antral stage, where FSH-dependent survival, AMH secretion, and inhibin B production are established. Follicles failing to acquire sufficient intraovarian growth factor support undergo preantral attrition governed by the PI3K/AKT/BCL-2 pro-survival balance before gonadotropin dependence is established. In sheep, KIT ligand specifically promotes the primordial to primary follicle transition; analogous PTEN and FOXO3 expression has been characterized in the prenatal and postnatal human ovary. Solid black up arrows – increase; solid black down arrows – decrease; solid black horizontal arrows – signal flow; solid red line – quiescent pathway; sold green line – activation pathway; solid blue line – primordial to primary follicle conversion transition to antral follicle; red dashed lines: attrition; greed dashed lines - self feedback loop. PTEN, phosphatase and tensin homologue; PI3K, phosphatidylinositol 3-kinase; AKT, protein kinase B; PIP3, phosphatidylinositol-3,4,5-trisphosphate; FOXO3, forkhead box O3; KITL, KIT ligand; BMP4/7, bone morphogenetic protein 4/7; SMAD1/5/8, mothers against decapentaplegic homologue 1/5/8; IGF-1, insulin-like growth factor 1; IGF1R, IGF-1 receptor; AMH, anti-Müllerian hormone; AMHR2, AMH receptor type 2; GDF9, growth differentiation factor 9; BMP15, bone morphogenetic protein 15; FSHR, FSH receptor; FSH, follicle-stimulating hormone; BCL-2, B-cell lymphoma 2.

The reproductive lifespan of female mammals is ultimately determined by the size of the primordial follicle pool established during fetal development and its progressive, irreversible depletion across postnatal life. Despite interspecies differences in the timing of follicle assembly, the core developmental sequence, oogonial proliferation, entry into meiotic arrest, and enclosure by pre-granulosa cells, is highly conserved across mammals (112, 113). Once established, the primordial follicle pool is maintained in long-term quiescent state through a balance of inhibitory and activating intraovarian pathways (**Figure 3**). Among the most important inhibitory regulators are AMH and phosphatase and tensin homologue (PTEN)/PI3K/AKT signaling, which together restrain premature follicle recruitment (25, 114). Conversely, activation cues such as KIT ligand, BMP4/7, and local growth factor signaling promote transition into the growing follicle pool (114, 115).

Primordial follicle quiescence is maintained through PTEN-mediated suppression of PI3K/AKT signaling, which retains FOXO3 in its nuclear transcriptionally active state to drive cell cycle arrest gene expression in the oocyte; once this inhibitory tone is overcome, follicular activation is irreversible (114, 116). Transition from quiescence to active growth is initiated by KIT ligand signaling through its receptor c-KIT on the oocyte surface, which activates PI3K to generate PIP3, driving AKT-mediated phosphorylation and cytoplasmic sequestration of FOXO3 and thereby relieving transcriptional repression of cell cycle progression genes in the oocyte (117, 118). Concurrent activation by BMP4 and BMP7, signaling through SMAD1/5/8 transcriptional complexes in pre-granulosa cells, coordinates somatic cell proliferation and enclosure around the activating oocyte, while locally derived insulin-like growth factors amplify PI3K/AKT signaling intensity to establish a self-reinforcing activation threshold that commits individual follicles to the growing pool (115, 117, 119). As activated follicles progress through the preantral stages, granulosa cell FSH receptor expression is progressively acquired through a gonadotropin-independent transcriptional program driven by oocyte-derived GDF9 and BMP15 signaling, establishing the molecular prerequisite for subsequent FSH responsiveness and marking the transition from intraovarian growth factor-dependent to systemic gonadotropin-dependent follicular development (115, 117, 120). Throughout preantral development, follicular fate is continuously determined by the balance between pro-survival PI3K/AKT/BCL-2 signaling and pro-apoptotic pathway activation, with inadequate intraovarian growth factor support triggering caspase-mediated granulosa cell death and autophagy-associated oocyte elimination that collectively drive the progressive attrition of the activated follicle cohort before gonadotropin dependence is established (118, 121).

Across mammalian species, AMH produced by granulosa cells of small growing follicles has emerged as the most reliable endocrine proxy of ovarian reserve, reflecting the functional size of the early growing follicle cohort and indirectly the remaining primordial follicle pool (25, 27). AMH participates actively in regulating follicle recruitment dynamics by suppressing FSH sensitivity at early antral stages, functioning as a negative feedback brake that calibrates the rate of follicular progression into the gonadotropin-dependent pool in proportion to the size of the remaining reserve (25).Ovarian aging is accompanied by coordinated endocrine remodeling characterized by rising circulating FSH, declining inhibin B, and progressive reduction in AMH secretion, reflecting diminishing granulosa cell mass and weakened negative feedback to the pituitary (28, 122). In cattle, similar aging-associated endocrine patterns include elevated baseline FSH, reduced inhibin A, and decreased antral follicle counts, closely paralleling reproductive senescence in women (121). These conserved trajectories indicate that ovarian aging is driven by shared regulatory principles rather than species-specific mechanisms. Longitudinal studies in dairy cattle further demonstrate that AMH concentrations measured early in life predict future reproductive performance (113, 123). These conserved endocrine trajectories reflect shared upstream regulatory principles of primordial follicle pool activation, preantral attrition, and gonadotropin-dependent selection across mammalian species, positioning cattle as a directly translatable model of human ovarian reserve decline and reproductive aging.

### 7.2 Oocyte quality decline and translational relevance to premature ovarian insufficiency

Declining fertility with age reflects not only quantitative depletion of follicles but also qualitative deterioration of oocyte competence. As oocytes age, cohesin proteins that hold homologous chromosomes together during the decades-long meiotic arrest gradually deteriorate, leading to errors in spindle formation, chromosome misalignment, and a progressive increase in aneuploidy risk (124, 125). Mitochondrial dysfunction represents another central mechanism of oocyte aging, characterized by reduced ATP production, increased oxidative stress, and altered mitochondrial DNA integrity. In bovine models, maternal aging has been directly associated with decreased oocyte ATP content and altered mitochondrial clustering patterns (126). These changes are accompanied by transcriptomic reprogramming in both oocytes and surrounding cumulus cells, impairing cytoplasmic maturation and developmental competence (127, 128).

Beyond structural and bioenergetic deterioration, oocyte aging is accompanied by progressive epigenetic drift that fundamentally compromises the transcriptional fidelity required for developmental competence. In bovine oocytes, genome-wide DNA methylation profiling using epigenetic clock methodology has revealed systematic age-associated hypermethylation at regulatory regions governing meiotic resumption and embryonic genome activation, providing a molecular correlate of age-dependent decline in oocyte quality that operates independently of follicle depletion kinetics (129). Ribosomal DNA loci represent a particularly vulnerable epigenetic target, with age-associated hypermethylation at these regions potentially impairing ribosomal biogenesis and translational capacity during embryonic genome activation (130). Physiological stressors accelerate this epigenetic deterioration through distinct but convergent mechanisms: metabolic perturbations during early lactation alter CpG methylation at loci regulating oocyte competence and embryo development (131), while thermal stress during maturation disrupts histone modification homeostasis and chromatin remodeling at meiotic checkpoints, increasing susceptibility to oxidative stress-mediated epigenetic damage (132). Extended *in vitro* maturation independently induces locus-specific methylation changes at imprinted gene regulatory regions in bovine oocytes, demonstrating that epigenetic vulnerability reflects a broader fragility of methylation maintenance mechanisms during the prolonged meiotic arrest characteristic of mammalian oogenesis (133–136). Age-associated deterioration extends beyond the oocyte to encompass the surrounding somatic cell compartment, with bovine granulosa cells exhibiting altered gene expression profiles and follicular fluid compositional changes that reflect progressive functional senescence of the follicular unit as a whole (137, 138). In women, granulosa cell DNA methylation patterns function as measurable indicators of biological aging pace and reproductive health trajectory, with forkhead box protein (FOXP)1-mediated epigenetic silencing of proliferative and steroidogenic gene networks representing a conserved mechanism of somatic cell senescence that directly undermines the follicular microenvironment supporting oocyte developmental competence (139, 140).

Reproductive aging in sheep and goats closely mirrors the human menopausal transition, with sequential depletion of primordial follicles, reduced follicular recruitment, rising gonadotropins, and eventual cessation of cyclicity (113). Genetic perturbations in oocyte-derived signaling pathways further reinforce the importance of intraovarian communication in maintaining ovarian longevity. Mutations in BMP15, GDF9, and related TGF-β superfamily members result in accelerated follicular depletion and infertility phenotypes resembling premature ovarian insufficiency (POI) in women (74, 141). Finally, livestock models have contributed directly to the development of fertility preservation strategies. Ovarian tissue cryopreservation, follicle isolation, and transplantation protocols were refined in large-animal systems before clinical translation and are now applied in women undergoing gonadotoxic treatments (142, 143).

## 8 Newer technologies bridging animal and human research

### 8.1 Ultrasonography and longitudinal follicle mapping

The introduction of real-time transrectal ultrasonography in cattle in the 1980s fundamentally transformed ovarian physiology by enabling non-invasive, longitudinal visualization of individual follicles across the estrous cycle. This methodological advance led directly to the identification of follicular waves *in vivo* and established a new conceptual framework for folliculogenesis as a dynamic, cohort-based process (2, 144). Beyond structural mapping, ultrasonography enabled integration of morphology with endocrine state, allowing direct temporal coupling between follicular dynamics and circulating gonadotropins and steroids.

The subsequent incorporation of Doppler ultrasonography extended this framework into functional vascular physiology. In cattle, follicular and luteal blood flow correlates strongly with steroidogenic output, particularly progesterone production in the corpus luteum, reflecting tight coupling between angiogenesis and endocrine function (145, 146). This vascular–endocrine coupling has been translationally extended to human assisted reproduction, where luteal and endometrial perfusion are increasingly recognized as correlates of implantation potential and IVF success (48, 147).

### 8.2 Superovulation, IVF, and embryo transfer: translational foundations

Embryo transfer in cattle, first refined in the 1970s and standardized in subsequent decades, provided the first robust demonstration that mammalian embryos could be recovered non-surgically, evaluated morphologically, and transferred between individuals while retaining full developmental competence (148). This established foundational principles of reproductive biotechnology, including embryo grading systems, synchronization of recipient endocrine environments, and controlled luteal support. Synchronization protocols were built upon controlled manipulation of progesterone and prostaglandin signaling to regulate follicular emergence and ovulation timing. The subsequent refinement of IVF in cattle, particularly through ovum pick-up–derived oocytes and *in vitro* maturation systems, extended these principles into fully ex vivo reproductive systems. Optimization of follicular wave synchronization, FSH-driven superstimulation, and suppression of premature LH surges using GnRH agonists or antagonists emerged directly from mechanistic insights into bovine ovarian physiology (26, 149, 150). These same principles underpin contemporary human IVF practice, where controlled ovarian hyperstimulation is fundamentally based on coordinated recruitment of a follicular cohort, suppression of endogenous LH surges, and controlled maturation of multiple dominant follicles.

### 8.3 Molecular profiling and single-cell approaches

The abundance of precisely staged ovarian structures accessible from domestic species through repeat ultrasonographic examination, particularly cattle and sheep, provides a molecular resolution of ovarian cell biology that is not achievable through opportunistic human tissue sampling or rodent models with fundamentally different reproductive physiology (4). Bulk transcriptomic analyses in cattle and pigs have defined stage-specific gene networks governing follicle recruitment, selection, dominance, deviation, and luteinization, revealing coordinated transitions in steroidogenic enzyme expression, growth factor signaling, and cell survival pathway activation that collective determine follicular fate (151, 152). Single-cell RNA sequencing has substantially refined this framework by resolving ranscriptional heterogeneity within granulosa and theca cell compartments, identifying functionally distinct subpopulations with stage-specific transcriptional identities that bulk approaches obscured through population-level averaging (153, 154). Cross-species single-cell transcriptomic comparisons between cattle, sheep, horses, and humans have revealed that core granulosa cell transcriptional programs governing steroidogenesis, gonadotropin responsiveness, and apoptotic regulation are broadly conserved, while theca cell androgen biosynthetic pathways, stromal inflammatory signaling, and granulosa cell senescence signatures exhibit greater interspecies divergence (155, 156). Integration of bovine and ovine single-cell datasets with human ovarian cell atlases, including follicular remodeling maps and cortical tissue profiles, has enabled alignment of conserved granulosa subpopulation states across species, identifying shared transcriptional regulators of follicular activation, oocyte-somatic communication, and luteinization while revealing species-restricted expression of specific receptor subtypes and paracrine signaling components (157–159). In sheep, integrative single-cell analysis has further mapped transcriptional programs governing follicular activation, oocyte maturation, and somatic cell differentiation across developmental stages, identifying stage-specific regulatory nodes that link intraovarian signaling networks to reproductive trait variation (159).

Genome-wide association studies have independently converged on many of the same pathways identified transcriptomically, with loci for age at menopause and ovarian reserve implicating DNA repair, homologous recombination fidelity, and immune-mediated follicular clearance as determinants of reproductive lifespan, consistent with single-cell identification of DNA damage responses in aging human oocytes and FOXP1-associated granulosa cell senescence (140, 160–162). The regulatory non-coding RNA landscape adds a further layer of post-transcriptional control to follicular development, with microRNAs expressed in bovine and human granulosa cells targeting gonadotropin receptor signaling, steroidogenic enzyme expression, and apoptotic pathway components in a stage-dependent manner that mirrors the transcriptional transitions identified through bulk and single-cell approaches (163–165). Long non-coding RNAs functioning as competing endogenous RNAs competitively sequester microRNAs through shared binding sites, thereby de-repressing target mRNA translation and modulating granulosa cell proliferation, steroidogenesis, and apoptotic signaling networks that collectively govern follicular fate decisions (164, 166). Beyond granulosa cell regulation, oocyte-derived non-coding RNAs transferred to surrounding cumulus cells through transzonal projections and gap junctions contribute to the coordination of oocyte-somatic communication, with bovine models demonstrating that this transcriptomic crosstalk regulates cumulus cell metabolic programming and developmental competence during follicular maturation (167). In horses, transcriptomic profiling across reproductive aging has identified conserved signatures of granulosa cell senescence and altered steroidogenic capacity (168).

Parallel advances in human ovarian single-cell atlases have identified DNA damage responses in aging oocytes and FOXP1-associated regulation of granulosa cell senescence, linking molecular aging signatures to functional decline in reproductive capacity (140). Complementary proteomic and metabolomic analyses of bovine follicular fluid have identified stage-specific metabolites, peptides, and growth factors associated with oocyte developmental competence, several of which correlate with fertilization outcomes and embryo quality in human IVF, reinforcing the translational validity of bovine follicular fluid composition as a proxy for the human periovulatory intrafollicular microenvironment (169, 170). Together, these converging molecular approaches are progressively reframing ovarian follicular biology from descriptive gene expression catalogues into mechanistically integrated regulatory networks governing follicular fate, oocyte competence, and reproductive aging.

### 8.4 Mechanistic studies not feasible in clinical settings

Large-animal models enable experimental approaches that are not ethically or practically feasible in human subjects. In cattle and sheep, periovulatory sampling at high temporal resolution permits characterization of rapid endocrine, transcriptional, and vascular changes induced by the LH surge (83, 171). In horses, intrafollicular injection and aspiration techniques enable direct interrogation of local signaling pathways during final follicular maturation (172). Targeted neuroendocrine manipulations in domestic species have further enabled causal dissection of hypothalamic control systems. Pharmacological modulation of kisspeptin signaling alters GnRH and LH secretion *in vivo*, directly validating its role as a key upstream regulator of reproductive axis activation (57, 173). *In vivo* electrophysiological recordings have further characterized state-dependent activity patterns in hypothalamic reproductive networks, linking neuronal firing dynamics to endocrine outputs (174). Controlled hormone delivery using chronic cannulation systems allows precise experimental control of GnRH pulse frequency and amplitude, enabling direct testing of the physiological requirement for pulsatility (175, 176). Together, these experimental platforms provide a level of causal inference in reproductive endocrinology that is not attainable in human systems.

## 9 Discussion

Fundamental mechanisms regulating ovarian function are highly conserved across mammals, encompassing the organizational logic of the HPO axis, follicular wave dynamics, oocyte–somatic cell signaling, steroidogenesis, luteal function, and ovarian aging (13, 83). The discovery of follicular waves in cattle (6, 144) established a discontinuous, cohort-based model of folliculogenesis, later confirmed in women through longitudinal ultrasonography (7, 23). This convergence defined follicular recruitment as a conserved mammalian process governed by endocrine cycling. In parallel, the requirement for pulsatile GnRH secretion for gonadotropin release was first demonstrated in animal models and subsequently validated in primates (8, 177). These conserved modules support the interpretation of domestic species as mechanistic models of ovarian physiology. However, translation requires careful consideration of species-specific differences in luteolytic signaling, ovarian vascularization, and seasonal reproductive programming (52, 53), which are listed in **Table 1**.

**Table 1:** Conservation and divergence of follicular and intraovarian mechanisms across domestic species and women.

Mechanism	Cattle	Sheep	Horse	Pig	Human	Translational equivalence	Clinical implications	Refs
Follicular wave dynamics	2–3 waves/cycle; single dominant follicle per wave; estradiol/inhibin feedback suppression	Reduced selection stringency; multiple dominants; BMP15/GDF9 thresholds govern ovulation rate	Extended dominant follicle persistence; prolonged LH/IGF support; single ovulation	Multiple follicles reach preovulatory size; reduced hierarchical suppression	2–3 waves/cycle; single dominant follicle; conserved FSH-driven cohort recruitment	Conserved organizational architecture across monovulatory species; ovulation rate divergence reflects quantitative BMP15/GDF9 signaling threshold shifts	Wave dynamics conservation informed controlled ovarian stimulation design; species differences in selection stringency explain FSH dose titration requirements	(1–3, 7, 11, 12, 18, 22, 23, 26, 27)
Two-cell steroidogenesis	LH-driven androgen synthesis; FSH-driven aromatization; IGFBP-regulated IGF bioavailability amplifies gonadotropin signaling	Conserved; IGFBP regulation characterized; BMP15/GDF9 modulate granulosa FSH responsiveness	Conserved; unique CYP17A1 isoform generates equilin and equilenin	Conserved; greater granulosa steroidogenic autonomy; reduced theca–granulosa interdependence	Conserved; DHEA contributes via peripheral aromatization	Highly conserved pathway organization; species differences are quantitative in enzyme activity and IGF bioavailability rather than architectural	Validates ruminant steroidogenic models for human anovulatory disorders; theca CYP17A1 hyperactivity and aromatase insufficiency mechanisms directly translatable	(3, 12, 20, 78–81)
Oocyte–somatic communication	Best characterized bovine model; cumulin heterodimer potency established; oocyte suppresses premature luteinization	BMP15/GDF9 mutations: heterozygous increases ovulation rate; homozygous causes follicular arrest, dose-dependent hierarchy control	Transzonal projection communication conserved; paracrine signaling less experimentally characterized	Conserved GDF9/BMP15; cumulin heterodimer characterized; granulosa autonomy reflects polyovulatory strategy	Conserved TGF-β/SMAD signaling; BMP15/GDF9 rare variants associated with primary ovarian insufficiency (POI) and altered ovarian reserve	Highly conserved at molecular level; sheep genetic models provide direct causal evidence for dose-dependent oocyte-derived control of follicular hierarchy	Sheep BMP15/GDF9 mutation phenotypes validate rare variant associations with POI in women, supporting clinical genetic screening strategies	(3, 11, 12, 68–71, 73–77)
Primordial follicle activation	Irreversible once initiated; PTEN/FOXO3 and KITL/c-KIT pathway well characterized in cattle	KITL promotes primordial to primary transition; BMP15/GDF9 mutations link dysregulation to POI	Conserved activation pathway; molecular characterization less advanced than ruminants	Conserved PI3K/AKT activation; reduced hierarchical suppression consistent with polyovulatory strategy	PTEN and FOXO3 expression characterized in prenatal and postnatal human ovary	Highly conserved PTEN/PI3K/AKT/FOXO3 architecture; BMP4/7 and KITL act on distinct cellular targets, pre-granulosa and oocyte respectively, across all species	Domestic species pathway characterization informed *in-vitro* activation protocols for fertility preservation in women with diminished ovarian reserve	(11, 24, 73–75, 114, 115, 119)
Ovarian aging trajectories	Progressive decline in antral follicle count, AMH, inhibin A; rising FSH; reduced oocyte ATP; genome-wide DNA methylation drift documented	Sequential primordial follicle depletion mirrors menopausal transition; BMP15/GDF9 mutations accelerate depletion resembling POI	Conserved granulosa senescence signatures and altered steroidogenic capacity identified through transcriptomic profiling	Endocrine aging trajectories less systematically characterized than ruminants	Rising FSH, declining inhibin B and AMH; oocyte aneuploidy through cohesin deterioration; mitochondrial dysfunction and epigenetic drift	Conserved endocrine trajectories and epigenetic drift reflect shared primordial follicle pool activation and preantral attrition principles	Longitudinal AMH protocols and epigenetic aging models refined in cattle directly inform clinical ovarian reserve monitoring and oocyte quality decline in women of advanced reproductive age	(113, 122–126, 141, 168, 169)

Seasonally breeding species such as sheep provide a particularly powerful example of reversible hypothalamic plasticity. Photoperiod-driven melatonin signaling modulates KNDy neuronal activity, suppressing GnRH pulsatility during anestrus and restoring reproductive function during the breeding season (66, 174). Genetic studies in sheep further reinforce the conservation of intraovarian signaling mechanisms. Mutations in BMP15 and GDF9 produce dose-dependent effects on ovulation rate and follicular hierarchy (11, 73), while analogous variants in humans are associated with diminished ovarian reserve and primary ovarian insufficiency (75). Spontaneous reproductive pathologies in livestock further enhance translational relevance. Cystic ovarian disease in dairy cattle represents a naturally occurring neuroendocrine–metabolic disorder characterized by disrupted LH surge generation (91). The prenatal androgenization model in sheep and cystic ovarian disease in cattle are referenced here primarily for their neuroendocrine and mechanistic contributions (**Table 2**), consistent with the scope of current review.

**Table 2:** Conservation and divergence of neuroendocrine and luteal mechanisms across domestic species and women.

Mechanism	Cattle	Sheep	Horse	Pig	Human	Translational equivalence	Clinical implications	Refs
Neuroendocrine GnRH pulsatility	Arcuate nucleus pulse generator; continuous GnRH causes receptor desensitization	Best characterized model; direct portal blood sampling enables precise temporal pulse characterization	Conserved; prolonged inter-pulse intervals during seasonal anestrus	Conserved; limited hypothalamic experimental access	Conserved requirement; hypothalamic circuitry less accessible *in vivo*	Fundamental conserved principle first demonstrated in animal models and validated in primates; species pulse frequency differences are quantitative rather than mechanistic	Enabled pulsatile GnRH pump therapy for hypothalamic infertility and GnRH agonist/antagonist protocols in ART	(3, 8, 28, 32, 33, 112)
KNDy oscillator function	GnRH pulse generator; disrupted in cystic ovarian disease through metabolic stress and altered steroid feedback	Best characterized model; pharmacological and *in vivo* electrophysiological validation of NKB/dynorphin oscillatory mechanism	Melatonin-mediated seasonal kisspeptin suppression; sequential reactivation at breeding season onset	Conserved; kisspeptin/GPR54 signaling confirmed	Loss-of-function mutations cause hypogonadotropic hypogonadism; KNDy dysfunction in functional hypothalamic amenorrhea	Highly conserved network architecture; sheep uniquely tractable for causal *in vivo* dissection of NKB and dynorphin contributions	Kisspeptin receptor agonists in clinical trials for hypothalamic infertility; NKB antagonists under investigation for menopausal symptom management	(9, 10, 52, 57–59, 65–67, 174, 178, 179)
Metabolic regulation of HPO axis	Negative energy balance during early lactation suppresses LH pulsatility	Nutritional restriction suppresses GnRH pulse frequency; models reversible nutritional anestrus	Seasonal and nutritional regulation through KNDy network; body condition directly influences cyclicity	Conserved; insulin and IGF-1 modulate hypothalamic sensitivity	Leptin, insulin, IGF-1 modulate arcuate kisspeptin drive and GnRH pulsatility	Highly conserved leptin-kisspeptin-GnRH metabolic sensor axis; species differences reflect quantitative energy deficit thresholds rather than mechanistic divergence	Dairy cow negative energy balance directly translatable to hypothalamic amenorrhea in energy-deficient women; shared kisspeptin suppression mechanism	(61–64)
LH surge mechanism	AVPV/POA kisspeptin surge mechanism; impaired in cystic ovarian disease	Best characterized model; estradiol-responsive AVPV/POA kisspeptin neurons directly validated; presumptive participation of Arcuate KNDy neurons; seasonal suppression reversible without loss of surge capacity	Prolonged preovulatory LH rise rather than discrete surge; sustained kisspeptin activation under extended follicular dominance	Discrete surge; estradiol positive feedback conserved; neuroendocrine characterization less advanced	Conserved; hypothalamic circuitry less accessible *in vivo*	Conserved kisspeptin mechanism; mare surge kinetics reflect quantitative LH secretory differences rather than mechanistic divergence; incomplete hypothalamic access in women is the primary translational gap	Kisspeptin-based ovulation induction validated through large animal models; GnRH agonist trigger in ART exploits conserved positive feedback mechanism	(2, 3, 9, 21, 52, 53, 57–59, 66, 180)
Luteolysis signaling	Pulsatile uterine PGF2α via FP receptor cascade; progressive LH receptor decline, cAMP impairment, steroidogenic enzyme downregulation, and apoptosis	Conserved PGF2α-driven luteolysis; oxytocin-PGF2α positive feedback amplifies signal via uterine-ovarian countercurrent transfer	PGF2α present; countercurrent transfer less prominent due to vascular anatomy differences	Estrogen-mediated uterine PGF2α receptor upregulation is primary initiating mechanism; distinct from ruminants	LH withdrawal combined with immune cell infiltration, cytokine signaling, and tissue remodeling; less strictly PGF2α-driven	Initiating signals diverge substantially between ruminants and women; terminal intracellular endpoints, reduced StAR, mitochondrial dysfunction, and apoptosis, conserved across all species	PGF2α initiation divergence limits translation of ruminant luteolysis models to human luteal support; conserved terminal endpoints validate ruminant models for luteal regression mechanisms relevant to ART luteal phase optimization	(3, 83, 84, 89–91)

### 9.1 Knowledge gaps and future directions

Despite substantial progress, key mechanistic gaps remain. First, the precise neural circuitry governing the preovulatory LH surge in women remains incompletely accessible *in vivo*, limiting direct mechanistic validation of hypothalamic models. While large-animal systems provide valuable proxies, species differences in hypothalamic organization and steroid feedback sensitivity constrain full translational equivalence (52, 57). Second, determinants of inter-individual variation in primordial follicle pool size and activation dynamics remain poorly defined, despite conservation of PI3K/AKT-mediated follicle activation pathways across mammals (24, 119). Third, integration of multi-omics datasets across species remains methodologically challenging, although emerging single-cell and comparative transcriptomic frameworks enable partial cross-species alignment of ovarian cell states (159, 181). Fourth, the transition from gonadotrophin-independent to gonadotrophin-dependent follicular development remains a fundamental unresolved problem in ovarian biology, for which domestic species, particularly cattle and sheep, provide uniquely tractable systems (4, 15). Future progress will require coordinated interdisciplinary approaches integrating reproductive endocrinology, neurobiology, comparative physiology, and systems biology.

### 9.2 Conclusions

Domestic animal species have been central to modern reproductive biology, enabling discovery of follicular wave dynamics, elucidation of oocyte-derived signaling systems, characterization of hypothalamic pulse generation, and development of assisted reproductive technologies. The conservation of ovarian regulatory mechanisms across mammals provides a unified mechanistic framework for understanding fertility, reproductive aging, and ovarian dysfunction across species. Continued bidirectional integration of animal models with human clinical research and multi-omics datasets will be essential for advancing mechanistic understanding and improving fertility preservation, reproductive health, and therapeutic intervention strategies across the lifespan.
